# From Project Bluestone to Operation Soteria Bluestone: An Academic-Police Collaboration

**DOI:** 10.1007/s43576-022-00065-y

**Published:** 2022-10-05

**Authors:** Elizabeth A. Stanko, Sarah Crew

**Affiliations:** 1 University College London, City, University of London, London, UK; 2 Avon and Somerset Police, Bristol, UK

**Keywords:** Rape, Sexual assault, Policing, Police reform, Justice response

## Abstract

The UK Home Office commissioned a government review of the criminal justice response to rape and serious sexual offences in March 2019 to explore for adult offences criminal justice outcomes, such as the proportion of cases being charged, prosecuted, and convicted in England and Wales. The report boldly concluded that ‘too many rape victims do not receive the justice they deserve’ (George and Ferguson in: Review into the criminal justice system response to adult rape and serious sexual offences across England and Wales: Research report, HMG, 2021, p 3). Launching Operation Soteria as a response to this review, the UK Home Office pledged to increase the number of rape cases making it to court. This contribution is a policy paper which was developed by capturing reflections from [Professor Betsy Stanko captured by reflections from Sarah Crew, and herself—denoted as S below], Chief Constable, Avon and Somerset Police, National Police Chiefs Council Lead for the policing of Adult Rape and Serious Sexual Offences England and Wales, and [Professor Betsy Stanko captured by reflections from Sarah Crew, and herself—denoted as B below], Strategic Advisor and Academic Lead, Project Bluestone and Operation Soteria Bluestone. They consider how this approach to improving the justice response to rape came about, bringing together academics and police forces across the country.

## Introduction

The UK Home Office commissioned a government review of the criminal justice response to rape and serious sexual offences (RASSO) in March 2019 to explore for adult offences criminal justice outcomes, such as the proportion of cases being charged, prosecuted, and convicted in England and Wales. The report boldly concluded that ‘too many rape victims do not receive the justice they deserve’ (HMG, 2021, p. 3).

Launching Operation Soteria as a response to this review, the UK Home Office pledged to increase the number of rape cases making it to court. According to the Home Office, the Operation Soteria programme objectives are to:

1. Test a series of tools (such as improved approaches for digital capture) and techniques (such as those associated with offender management) to engender justice outcomes. As well as potentially increasing charge rates, the tools and techniques will enable the proactive making of applications for civil orders, such as sexual risk orders; and
2. Develop a national operating model (created through an evidence-based collaborative project) for better and faster rape investigations that can be taken up by police forces and Criminal Prosecution Service (CPS) areas from 2023.

The articles in this symposium discuss the theory-inspired, empirically led framework from which the improvement in the policing of rape and sexual assault is founded. We set out a preview of the kinds of data, scholarly scrutiny, and collaborative co-production mechanisms for changing police practice. Hohl and Stanko (this issue) set out the theoretical ‘five pillars’ framework that underpins Operation Soteria Bluestone. The project is intentionally designed to be thoroughly collaborative between academics and police officers. Each of the pillars outlined in Hohl and Stanko has an academic lead; each of the police forces has a police practitioner lead. Together they create a bespoke approach to improving investigations. At the same time, the purpose of the project—to create a national model for the investigation of rape and sexual assault—melds the learning, the analysis, and a shared vision that is agreed between the academic teams and the police teams.

In England and Wales, senior officers lead portfolios of police practice on behalf of the National Police Chiefs Council. These senior officers steer improvement in police practice, advocate for legislative change, and act as the police voice in the national conversations representing policing in the substantive areas they lead. This contribution is a policy paper developed from a conversation held between Sarah Crew, Chief Constable, Avon and Somerset Police, National Police Chiefs Council Lead for the policing of Adult Rape and Serious Sexual Offences (RASSO) England and Wales, and Betsy Stanko, Strategic Advisor and Academic Lead, Project Bluestone and Operation Soteria Bluestone. They consider how this approach to improving the justice response to rape came about, bringing together academics and police forces across England and Wales. In this policy piece, we get a glimpse of the project’s inception, the rationale behind trusting this approach to improvement policing and the difference police practitioners think this unique collaboration between academics and police practitioners is making to policing, and the justice outcomes for victims of rape and sexual assault in England and Wales who report their crimes to police.

MOPAC^1^ reached out to (then) Deputy Chief Constable Sarah Crew about the academic underpinnings for Project Bluestone towards the end of 2019, and Betsy and Sarah first had a conversation about it in July 2020. Sarah had a meeting in early 2020 with colleagues in MOPAC before the Covid-19 lockdown was implemented, when she first heard about the work being done because of the London Rape Review. Sarah remembers the discussion resonating, but it being largely conceptual, and not seeing any paperwork until after the Violence Against Women and Girls Network Research Network Webinar in July 2020.^2^ She decided to collaborate and to support what came to be called Project Bluestone because it was taking ideas she already knew worked, and framing them in an evidence-based way, using a solid academic underpinning. She had gained this insight, having been involved with the original Bluestone iteration project, which was the creation of specialist rape investigation units in Avon and Somerset^3^ between 2010 and 2014. This approach was conducted without the academic foundation, but had a practitioner’s view of what worked in terms of focussing on suspects, supporting victims, and working as a team with different services. What she felt the new Project Bluestone did was to include the learning from this previous project, whilst also integrating the important factors including specialism, resilience of police officers, and procedural justice, as well as the importance of the underpinning force-held data used strategically. The five pillar approach seeks to codify, understand, and classify systematic ways of working which are founded in scientific approaches, and which Sarah acknowledges policing does not do routinely at the moment. Instead, policing is much more reactive to immediate events and manages the immediate situations with the aid of initial training, followed by intuitive decision making. To bring in a scientific discipline and framework, therefore, was one of the most important aspects of this project.

From the outset, what the core academic team really needed was a practical application; a place to trial the five pillar approach in the real world as the corporate tethers for improvement. The opportunity for this to happen was through some government STAR funding.^4^ Sarah held a conversation with her NPCC portfolio staff police officer about the project and noted at the time that improvements to the approach to rape and sexual offences investigations needed to be made and that this approach, now labelled by the team as Project Bluestone, was a mechanism to do just that. As noted above, the proposal had elements familiar to Sarah from previous research, and with the uplift^5^ in detectives Avon and Somerset were due to receive they had the resources to host the pilot project. The STAR funding provided a timely opportunity to progress the project and, in actual fact, the strict timelines dictated by the STAR bid^6^ was helpful in terms of fully focussing Avon and Somerset to work through potential barriers quickly to make sure the research project progressed in a timely manner. This included addressing the practical obstacles faced by academic researchers, such as obtaining vetting for researchers and putting data sharing agreements in place.^7^

One of the questions that is often asked of Project Bluestone is how the collaboration between academics and police practitioners has worked, in terms of the trust required from the police to bring the researchers in and provide open access to officers and data. Part of this was the understanding from Sarah that we are all professionals who were working towards a common goal. Having the starting point of a shared intent, it meant that all parties were willing to invest in the time it takes to build trust. The broader context of the ongoing Covid-19 pandemic also meant that what was orthodox practice in conducting policing research was being challenged. Working on site was impossible for the researchers, meaning that focus groups, interviews, and consultation were all completed virtually. Whilst the working world was changing, the project was provided with an opportunity to try alternative methods of conducting research by all parties for the public good. From Sarah’s perspective, she notes she never thought of doing anything other than trusting the researchers. Although she was well aware that they would find things that were uncomfortable to hear as a senior police officer, her rationale was that, with things being as bad as they were in the national justice outcomes for rape and other sexual assault, they couldn’t become much more uncomfortable. Sarah was a year into being the national NPCC lead for rape and serious sexual offences and had grown tired of defending police practice to stakeholders and government. Instead, she wanted to have something to say that was more positive and forward looking, and so Project Bluestone arrived for Sarah at just the right time.

In practice, researchers and police officers were careful to work within the law and the boundaries within their professional disciplines, but with a shared aim. The researchers all felt welcomed by officers and experienced a sense of openness which was quite remarkable. The Chief Constable of Avon and Somerset at the time is credited with facilitating the project by supporting Project Bluestone, the funding bids, and encouraging the open research to take place in his force. This is a timely reminder of the importance of not underestimating the power in a command-and-control organisation of a senior member of staff giving permission for a project like this to occur. Because Sarah was the National Lead for the Policing of Adult RASSO and the deputy Chief Constable, and because she had that senior support, she was able to take ownership of progressing project, and which subsequently gave everyone else permission to be open and to engage with the research. This further demonstrates the importance of internal, organisational champions for change. This lesson has subsequently been applied across the board to ensure there are senior champions in all the forces working with the project.

For the researchers, what was crucial about this project was that they were going to influence policing practice from within. But the process of translating the approach into findings and then producing useable recommendations and products is not often articulated in research. For this project, it was key to approach this as a whole research / police team. Each academic pillar lead was matched with a police pillar lead which enabled them to build up a good, professional, equal relationship which was based on constructive challenge. Additionally, the process was also iterative, meaning it was happening in real time. This was in contrast with many previous projects with academics and police where the findings are often produced and shared well after the research takes place. In fact, this happened with the evaluation of the original Bluestone units, whereby the research was published saying the specialist units were having a positive impact on justice *after* they had already been disbanded (Rumney et al., 2016). In this respect, the timeliness, the real-time iterative nature of the project, and the constructive challenge built into the team and created with both academics and practitioners were all critical to the success of producing useable outputs. This approach is not without challenges. The researchers had to have the commitment to work quickly, grappling with the challenge of implementation and the practical considerations this brought. Building the foundation in Avon and Somerset, however, including the timeframe and the relationships, enabled researchers to think about how the Bluestone model could be translated into the different forces they are now currently working with.

For all parties, when the project is complete it will be necessary to take time to reflect on how it has changed the academics’ and police officers’ working practices. For example, a Detective Inspector, the Pillar 2 lead in Avon and Somerset, has become quite focussed on using academic approaches in everyday policing work and how the project established a systematic way of working. There is a shared hope that there will be a lasting legacy of this project that is wider than the investigation of RASSO, which will be introduced into different components of working in a whole systems organisation. Sarah reports that, within Avon and Somerset, the senior team is thinking about how this approach can change police culture across the board, and the changes required in the use of behavioural and social science and organisational psychology to help police understand new ways of working. Avon and Somerset Police have already started to think about where they can access resources within academia, as opposed to liaising with large consultancy firms to solve institutional problems. Of course, finding common ground when conveying the research findings to senior officers can prove challenging, especially given the project’s complexity. For the researchers, this experience has impacted on our collective thinking about policing policy in England and Wales, and traditional academic approaches to influence policy. Perhaps surprisingly for Sarah, the wider learning was that there is currently a lot wrong in the world of policing! And improving justice does not stop at the police’s door; Sarah is clear that it is essential for the whole criminal justice system to be perpetrator focussed. She acknowledges that policing already works closely with other partners when it comes to prevention and rehabilitation, and the project is demonstrating that when it comes to policing criminally active people, policing should be able to come into its own expertise and ought in this instance to take the lead. Policing is demand-led, and the project has found that police resources focus too much on the assessing and investigating the credibility and actions of the victim alone, and less focussed on the people that commit sexual offences. The question, therefore, is how policing—and particularly the investigative function—can realign itself guided by research evidence, so that it better investigates those that commit crime and harm. That involves the police, as Sarah says, understanding how to disrupt, catch, and convict more widely, and at the same time working with other agencies to prevent and to divert offenders.

Another area Project Bluestone has been focussed on is attempting to influence the way the Government is thinking about the recent rape review (George & Ferguson, 2021). Both Sarah and Betsy have taken every opportunity to be completely upfront in this regard, emphasising the approach—perpetrator focussed investigations—which is at the heart of Bluestone as a whole. Bluestone is mentioned as promising practice by the UK government’s rape review because there were opportunities along the way to advocate, champion, and use this evidence base to argue for transformational change, using the ongoing project as a live example of a mechanism for that change. The opportunities to share the project’s methodology with a wider group of stakeholders who influence ministers, politicians, and policy makers ran parallel to the completion of the government’s review in Spring 2021, emphasising in particular the shared interest that the academics and Avon and Somerset had in iterative, creative solutions which brought about a change in practice. It was felt that, given the broad and shared interested amongst so many different groups and stakeholders in England and Wales who are rightly concerned about the appalling state of justice for rape and serious sexual assault victims, the project strongly influenced the proposed next steps for government policy on the investigation of RASSO. Part of this was about ensuring that the team were in the right place at the right time, when the right questions are being asked or the right discussion is being had, and to come forward with some evidence or an informed understanding that changes the way people think. Whilst these changes may not be immediate, the ideas then have an opportunity to take root, such as talking to other key police leaders such as the National Police Chiefs Council Lead for Violence and Public Protection and taking those opportunities to speak to ministers or officials in national and other forums. A good example of this type of engagement would be the Law Commission who engaged with Sarah recently as a result of the rape review, and the team as a whole have used both evidence informed narratives about victimisation and offending with data, to try and make people think differently based on the evidence of and about RASSO, planting seeds to eventually grow for people to alter victim-blaming narratives still rife in policing, with professional practice grounded in investigation strategies and documentation which explored issues of offending patterns and offending behaviour.

One barrier to conducting research on rape and sexual offending is access to data and intelligence directly from police reported crime. Project Bluestone has proved that it is possible to enable researchers to have access to aggregate police crime data even when there are issues of confidentiality and security to work through. We are all governed by the law in the UK and we have strong data protection laws. This means that, in principle, police forces should be comfortable with sharing these types of data, for research purposes, and is an ethos that Sarah agrees with. Often the reality in policing, however, is not a lack of will to data share, but often the logistical issue of how to get data out of what are typically difficult systems to work with. Sarah is of the opinion this is why police are not exploiting their own data properly, because the crime recording systems have changed and developed over time to accommodate various requirements which are often grounded in the tactical but not the strategic use of information. One of the benefits of Project Bluestone may well be the demonstration of the value of the data police hold and how it could be used. There may be other aspects of the criminal justice system where there are other sensitivities to consider, but speaking from a policing perspective, as long as the law is being followed, Sarah was confident that in this instance the data sharing could be managed through the use of strong data sharing agreements with strong oversight from the Information Commissioner’s Office. With this access, academics are then in a position to analyse data to demonstrate to the police how they can explore their own data to improve and to monitor their practice with an eye to continuous learning and improvement.

It is still, however, very unusual in the UK to have the kind of access that Project Bluestone researchers have had, and as the project is planning to combine the datasets from different forces it is likely these data will enable more insight about the barriers to justice on an unprecedented scale. From Sarah’s perspective this is seen as a great benefit because it means the research findings and the subsequent direction of travel for change have a strength that cannot be challenged when policy arguments are made using opinion and smaller datasets, rather than harnessing a more definitive and comprehensive database. In the criminal justice system, for example, relationships between internal stakeholders, such as the Crown Prosecution Service and the police service, suffers from debates either uninformed by data, or arguments about what disjunctured data may say about justice. The fact that the researchers are exploring issues at both an individual force and at a broader level means that some of these ongoing debates can focus on the important strategic issues that would lead to improvement in this area. The level of access Project Bluestone researchers have had on this project is hugely important and this, combined with the researchers as independent ‘brokers’, enables harder truths to be tabled. A key next step for Bluestone is to consider is how the approach and the findings can be shared nationally and internationally.

In order to implement the findings from Project Bluestone, researchers and the police need to overcome cultural challenges and resourcing issues. Sarah believes that, for the cultural shift that needs to happen, there are a lot of factors that need to change. The political attitude for change is always short-term. Keeping politicians and policy officials patient for change is taxing. Having said that, in England and Wales there is political will to make change at the moment, and this project must capitalise on that. In order to do this, as a collaborative team we need to be much more vocal in the public domain, talking about the journey we’re on and gaining people’s understanding and knowledge, because this will buy the time to make the cultural change within the police service that needs to happen. Clearly Bluestone’s influence goes beyond the policing culture of investigating rape and speaks more broadly to the culture of policing. This requires a deeper consideration about how police officers learn and develop over the course of their career, and this will hopefully have a knock-on effect of better integrating academic scholarship into policing practices in a timely manner.

In terms of other potential impacts of Project Bluestone, there are also likely to be effects on other agencies the police have contact with who work with both victims and offenders. From an Avon and Somerset perspective, for instance, Bluestone has already recalibrated a poor working relationship with the Crown Prosecution Service (CPS) into a positive, respectful one. It is too early to tell how much of that has simply come from individual commitment or organisational shifts. Bluestone’s approach has already seeped into ways of working for the CPS, and they participate in joint learning sessions with the police service. Sixteen months on, Bluestone has really changed that relationship, taking Avon and Somerset’s police-CPS relationship from tiptoeing around each other, to both agencies working together from a strategic point of view. Sarah is certain it is the evidence from Project Bluestone that has demonstrated to the CPS the value of working differently. Whether this can be replicated nationally and internationally remains to be seen.

Operation Soteria is a UK government sponsored and funded programme of work that includes Operation Soteria Bluestone (the police change component discussed above). The CPS is part of Operation Soteria and leading its own development and change. Developed conceptually and theoretically by academics, Operation Soteria Bluestone is grounded in social science methods, organisational nouse, and supported by leadership throughout the criminal justice system. Originally designed as a three-year project, the funding is year to year—precarious for those working in academic institutions where much longer planning is more common. As Sarah muses, it will be important to share the academic perspectives of what it has meant to work differently and in such a real-time, collaborative manner.

There are some key take away points from the above. First, leadership is critical to moving forward challenges to policing practice and culture. Senior champions who are in post long enough to stabilise improvement is also key. Trust—in academic knowledge and scientific analysis—shifts the foundation for traditional police decision making into professional decision making informed through academic scholarship. This is of course the aspiration of what is now referred to as evidence informed policy making. For the next steps of Operation Soteria Bluestone, plans are already underway to extend the project’s remit to more police forces. This stretches the kinds of academic support available to the police service more widely in England and Wales in the investigation of RASSO. This has put more pressure on the academic researchers, who feel more comfortable with steering practice with robust, academic analytic insight rather than serving as advisors to change. Evaluating the evolution and implementation of this national model for investigating RASSO is scheduled for 2023, and at the moment the academics involved in the project are steering both. At time of writing, it is hoped that the project will receive funding on a sustained basis so that the insight into the investigation of RASSO from a policing perspective can contribute solid lessons globally.

## Footnotes

1 Mayor’s Office for Policing and Crime, London. MOPAC published a report in July 2019 on the Metropolitan Police’s performance in investigating rape. A small team of colleagues from MOPAC and Stanko and Hohl (see this issue), wrote a short brief describing the project’s intent and approach, and began speaking to civil servants, police officials and key members of government, from September 2019 about undertaking the project as the mechanism for improvement to the police response to rape and sexual assault.

2 https://vawgnetwork.mdx.ac.uk/

3 Chief Constable Sarah Crew led the rape investigation unit [called Bluestone] in Avon and Somerset between 2010 and 2014.

4 The STAR fund is a bespoke source of UK Home Office funding for police projects linked to science, technology, and academic research.

5 The UK Home Office provided additional funding to police forces in 2019 to hire more police officers, as the overall staffing complement of police had been reduced due to government spending austerity over the past decade.

6 Serendipity played a key role in this project journey. The proposal was written within a week, using the brief already prepared for the high-level conversations in government. The proposal was submitted in October 2020, funded in January 2021 to be completed by end of March 2021.

7 The process of gaining access to police forces for the purposes of research is a belaboured, bureaucratic one. All researchers needed to be vetted by the police, and detailed data sharing agreements in place. Furthermore each university research participant is required to submit ethics panel applications for all research conducted under university sponsorship in the UK. These processes took place at record speed.
